# Imaginative Enrichment Produces Higher Preference for Unusual Music Than Historical Framing: A Literature Review and Two Empirical Studies

**DOI:** 10.3389/fpsyg.2020.01920

**Published:** 2020-08-21

**Authors:** Anthony Chmiel, Emery Schubert

**Affiliations:** ^1^The MARCS Institute for Brain, Behaviour and Development, Western Sydney University, Sydney, NSW, Australia; ^2^Empirical Musicology Laboratory, School of the Arts and Media, University of New South Wales, Sydney, NSW, Australia

**Keywords:** music preference, framing, contextual information, program notes, imagination quality, valence, historical

## Abstract

Does accompanying information (“framing”) such as in a program note influence our preference for music? To date the findings have been mixed, although a small body of research has suggested that when framing accompanies music considered unusual (characterized by extreme complexity and extreme unfamiliarity), the music may be preferred compared to when no such framing occurs. A literature review (study 1) revealed that for 50% of experiments where valenced framing (positive *versus* negative suggestions of prestige) was manipulated, positive framing was accompanied by significantly higher ratings of preference and/or quality judgements. However, only one example contained music that could be considered unusual (atonal music). We therefore conducted two follow-up experiments, with each examining the influence of valenced framing *as well as* historical framing (accompanying historical details) for music intended to be unusual. Study 2 manipulated framing for an excerpt using atonal music, although we were unable to find evidence that positively valenced historical framing increased preference for this piece. A surprising finding in study 2 was that our active control—requiring the participant to engage imaginatively with the music—produced a significant increase in preference. Subsequently, in study 3 we examined the same three framing conditions and included both an unusual excerpt (free jazz) as well as an over-familiar, typical excerpt for comparison (being a repeatedly pre-exposed classical piano piece). Study 3 produced no significant differences in preference ratings between the two historical conditions, although a positive impact of imagination was again evident. We concluded that the impact of historical framing may be highly subjective and not of favorable consequence to the typical listener. Furthermore, while imaginative engagement appears a fruitful avenue for further preference research, it has been largely ignored.

## Introduction

Music performances and sound recordings are often accompanied by information such as program notes, liner notes, commentaries, and reviews, but the question remains as to whether this information impacts our preference, and if so, to what extent. Accompanying information can be referred to as background information, context, or “framing”. “Historical framing” refers to historical information accompanying a work and specifically to studies in which the amount and/or type of historical information accompanying a work is manipulated. “Valenced framing” refers to manipulation of the purported quality or prestige of a work under consideration or could consist of positive *versus* negative framing such as the suggestion of favorable or unfavorable reactions to the work from the general public. Another example of valenced framing is when the effort purported to create the work is overstated, compared to understated. Overstated accounts, such as the time taken to create a work of art, have produced improved evaluations for the work in question (e.g., Kruger et al., 2004; Jucker et al., 2014).

The impact of framing on music preference has been empirically investigated since at least the 1930s (Damon, 1933), although no clear consensus has been reached. This indeterminacy has been highlighted by a recent review of the literature (Chmiel and Schubert, 2019a) concerning the impact of historical framing on preference for both music and visual works. Inspired by Bullot and Reber’s (2013b) *psycho-historical framework for the science of art appreciation* (henceforth PHF), Chmiel and Schubert’s review examined the hypothesis that the presence of historical framing should produce significantly different ratings of preference when compared to conditions receiving either no historical framing or less historical framing. Chmiel and Schubert reported heterogenous results, with the majority of the reviewed experiments (56%) concluding no significant difference in preference due to the presence of historical framing and 18% of experiments being inconclusive (that is, mixed results within the same experiment). In total, 26% of the examined cases reported a significant difference in preference due to historical framing; this produced a positive change in preference for 21% of the overall studies and a negative change in preference for the remaining 5%. One factor that was hypothesized to play a role in the effectiveness of framing upon preference is the so-called “unusualness”^1^ of the music, where preference for unusual music is facilitated by historical framing. The authors noted that this assertion has received little attention in the literature.

Literature concerning valenced framing has not been systematically reviewed in the same manner that literature concerning historical framing has. To further complicate this research area, studies concerning valenced framing often contain quality as *both* a dependent variable (i.e., participant ratings) as well as an independent variable [i.e., manipulated quality between conditions; for an example, see Kruger et al. (2004)]. Consequently, we make further distinctions between preference and quality as separate dependent variables, and also between quality as a rated dependent variable and quality when manipulated as an independent variable for valenced framing. Henceforth, in this paper, we use preference as an umbrella term for all aesthetic evaluations, such as appreciation, enjoyment, hedonic tone, liking, pleasingness, and so on. Similarly, quality as a dependent variable will be used as an umbrella term to extend to evaluations of value, prestige, or goodness. As an independent variable, quality will be used to refer to manipulation of associated value, prestige, or goodness, or for positive/negative framing (as outlined above, regarding purported audience responses). In this way, preference and quality are able to coexist as separate but related dependent variables within the same study, and two aspects of quality are able to coexist as dependent and independent variables within the same study.

The present paper reports a literature review (study 1) intended to complement and extend Chmiel and Schubert’s (2019a) review on historical framing by examining literature on valenced framing and music preference. Following this, we report two empirical studies examining the impact of three types of framing on music preference for unusual music. Specifically, study 2 manipulated framing for a single “unusual test piece”, being an excerpt of atonal music. Study 3 expanded the design by manipulating framing for both an unusual test piece as well as an over-familiar, typical-sounding test piece to better understand if low familiarity interacted with framing.

## Study 1 – Literature Review

### Method

To be included in the review, literature needed to report (a) ratings of preference (or an equivalent) as a dependent variable or (b) ratings of quality (or an equivalent) as a dependent variable or (c) ratings of both variables as dependent variables. Additionally, valenced framing (as an independent variable) needed to be manipulated in such a way that two or more conditions received different associations or presentations concerning quality, value, prestige, goodness, positive/negative framing, and the like^2^. Unlike Chmiel and Schubert (2019a), the included studies were limited to experiments containing music stimuli. Literature to be included was identified using various combinations of general and keyword searches, such as “preference” and “quality” (or an equivalent term, as outlined in the “Introduction” section) and “music”, “framing”, “context”, “background”, “program notes”, and the like. Searches were performed in *Google Scholar*, *Répertoire International de Littérature Musicale* (RILM), and *PsycINFO*. Articles cited in these papers were also assessed to encompass a larger number of possible papers that satisfied the inclusion criteria.

We then analyzed each experiment that met the inclusion criteria by placing them into one of the following three categories:

(A) An experiment in which the positive manipulation of valenced framing (such as a suggestion of increased value or quality) is accompanied by a statistically significant higher rating of preference. We set the criterion for “statistically significant” to comparisons that produced results which reject the null hypothesis with a type 1 error of less than 5% (i.e., *p* < 0.05, with corrected value if required), applying the same criterion across all studies, based on the relevant statistics reported by their authors or otherwise ascertained.
(B) An experiment reporting inconclusive results, that is, a mixture of results from categories “A” and “C” reported within the same experiment.
(C) An experiment in which the positive manipulation of valenced framing (hence “positive framing”) is accompanied by either a statistically significant lower rating of preference or no statistically significant results. The criterion for “statistically significant” was identical to category “A”.

### Results

Fourteen experiments met the inclusion criteria, taken from 12 separate publications. The experiment details and the categorizations for the included studies are listed in Table 1. The publications ranged in publication year from 1972 to 2018. Of the 14 experiments, seven (50%) were categorized as “A” (strictly significant positive differences in preference accompanying positive framing), five experiments (35.7%) were categorized as “B” (inconclusive results), and two experiments (14.3%) were categorized as “C” (either no significant differences in preference accompanying positive framing, or a significant decrease in preference accompanying positive framing). One of the two experiments categorized as “C” (Chapman and Williams, 1976, experiment 2) reported significantly lower ratings of goodness for a high status condition compared to a control, whereas the other experiment categorized as “C” (Steinbeis and Koelsch, 2009) reported no significant differences between conditions. Five experiments contained preference or an equivalent as the dependent variable, and six experiments contained quality or an equivalent as the dependent variable. Additionally, two experiments contained both preference and quality as separate dependent variables, and one experiment reported an aggregate score from ratings of both preference and quality as the dependent variable. We next examined categorization results split by dependent variable. Of the eight studies reporting results concerning preference, five (62.5%) were categorized as “A”, two (25%) were categorized as “B”, and one (12.5%) was categorized as “C”. Of the nine studies reporting results concerning quality, four (44.4%) were categorized as “A”, four (44.4%) were categorized as “B”, and one (11.1%) was categorized as “C”.

**TABLE 1:** Tabulation of literature reviewed on the influence of framing on preference and/or perceived quality of music.

Author(s), year, and experiment number^a^	Stimuli	*N*	Design and manipulation of framing	Label for dependent variable of interest	Result categorization^b^	Result details
Anglada-Tort and Müllensiefen, 2017	Elvis Presley’s “Jailhouse Rock” and Bruckner’s Symphony No. 4 (“Die Romantische”)	72	The participants heard each stimulus three times and were told that each exposure was a different performance. Each exposure was framed with a varying prestige level (low, medium, or high). For the Elvis piece, they were told that they would hear three Elvis impersonators and for the Bruckner piece that they would hear three different conductors	Liking	A	Linear mixed-effect analysis produced significant differences in condition for each piece. In both cases, the high-prestige condition rated liking higher than the medium- and low-prestige conditions. There were no significant differences between these latter two conditions
Aydogan et al., 2018	Identical to Kroger and Margulis (2017), exp. 1	20	Design is based on Kroger and Margulis (2017), exp. 1, except that all pairs of excerpts were heard a second time later on in the experiment, but this time with the professional/student labels reversed	Enjoyment	A	Wilcoxon signed-rank test indicated that enjoyment ratings were significantly higher when performances were framed as by a professional
Cavitt, 1997	A 74-s recording of a high school symphonic band performing a “beginner piece” titled “Westchester March”	41	The participants heard the same recording twice, although these were framed as performances from two separate bands (a 6^th^ grade beginner band with 7 months of experience and a high school band with 4 years of experience)	Quality	B	Between-subjects ANOVA produced a significant interaction between the band label and the (randomized) presentation order. Performances labeled “high school” were rated higher in quality when presented second but rated lower in quality when presented first
Cavitt, 2002	Two solo trumpet recordings of the first 25 bars of a Grade 1.5 concert band piece titled “Fanfare and Fugue”. Recordings were performed by a professional musician	39	One recording was intentionally created to be a “good” performance (in terms of tone and dynamic contrast) and the other to be a “bad” performance regarding these elements. Both versions contained “good accuracy”. The participants heard both recordings twice and were informed that these were by four separate 7^th^ grade students. The excerpts were matched with a description of the students’ ability and effort levels. Four conditions: high ability/high effort (good performance), high ability/low effort (good performance), low ability/high effort (bad performance), and low ability/low effort (bad performance)	Quality	A	Repeated-measures ANOVA produced significantly different levels of quality for performance type and also the described effort level. The quality ratings were significantly higher for the good recordings and were also significantly higher for the high-effort conditions (although the stimuli were identical between high- and low-effort labels)
Chapman and Williams, 1976, exp. 2	“Dorian Horizon for 17 Strings” by Toru Takemitsu, conducting the Toronto Symphony Orchestra	36	The participants were adolescents that liked progressive pop but did not like “serious music”. Split into three conditions. A high-status group was told that the piece was from a record by Roger Waters (Pink Floyd), who was positively framed as a progressive musician. A low-status group was given an accurate description of the piece, being classical music. A control was not given any information	Goodness	C	Between-subjects ANOVA produced a significant result of condition for goodness. Scheffé *t*-tests confirmed that the control rated goodness higher than both the high- and the low-status groups; there was no significant difference between these two latter groups
Duerksen, 1972	Beethoven’s piano sonata No. 6, 3^rd^ movement, performed by a world- renowned performer	517	Three conditions, exposed to an identical recording twice. For one group, the first excerpt was labeled as by a world-renowned performer and the second as by a graduate student. For another group, these labels were reversed. The control group was told that they would hear the same performance twice, with no additional information	Overall quality, and 6 musical parameters relating to quality	A	ANOVA showed that on all seven scales, the excerpt labeled as a world- renowned performer was rated significantly higher than the student-labeled excerpt. Additionally, the renowned excerpts were rated higher than the control excerpts
Fischinger et al., 2018	Mysliveček’s Sinfonia in E-flat major for two horns, oboes, and strings, 1^st^ movement (“Spirituoasa”)	170	A program note that described the composer, their background, and the music was used. A total of 86 participants were (correctly) told that the piece was composed by Mysliveček–described as a relatively unknown composer–whereas 84 participants were told that the piece was composed by Mozart	Liking	B	Between-subjects full-factorial ANOVA showed no significant main effect of composer but a significant interaction between composer and participant age. Younger participants (aged ≤ 40) rated liking significantly higher when the music was attributed to Mozart
Kroger and Margulis, 2017, exp. 1	90–120-s excerpts of eight piano pieces composed by Beethoven, Brahms, Chopin, Macdowell, Mozart, Say, Scarlatti, and Rachmaninoff	40	Each of the eight pieces had two versions–one performed by a renowned professional and the other by a conservatory student. In actuality, 50% of the time the participants heard the same version twice. The participants were asked to guess which version was by the professional and which was by the student	Enjoyment and quality	A	The participants were statistically more likely to label a version as professional when this was the case. The participants rated enjoyment and quality higher for the performance they guessed to be professional, even for identical excerpts
Kroger and Margulis, 2017, exp. 2	Identical to Kroger and Margulis (2017), exp. 1	40	Similar design to Kroger and Margulis (2017), exp. 1, except that one version was labeled as by a professional and the other as by a student. In actuality, some labels were incorrect, and at times the participants heard the same version twice	Enjoyment and quality	A	The participants were statistically more likely to prefer a version when labeled professional, regardless of the actual performer, or for two identical excerpts
Radocy, 1976, exp. 1	An excerpt from each of: Beethoven’s Sonata No. 8 in C minor, Op. 13 (2^nd^ movement), Brahm’s Academic Festival, and a cornet arrangement of “Sarabande” from Corelli’s Sonata VIII	150	Five conditions. The control received minimal instruction containing no bias toward any stimuli. M1 condition received information containing moderate positive bias toward their first presented excerpt, whereas M2 condition received information containing moderate positive bias toward their second presented excerpt. S1 and S2 conditions similarly received information containing strong positive bias toward the first and the second excerpts presented, respectively	Aggregate of 7 variables relating to quality	B	ANOVA showed a significant three-way interaction between stimulus, presentation order, and condition. Specific *post hoc* results are not reported, although the authors state that “differences among the bias conditions occasionally [were] non-significant and/or in the direction opposite to that expected” (p. 124)
Radocy, 1976, exp. 2	Eight excerpts: two baroque (Stoelzel and Quantz), two classical (Mozart and J. C. Bach), two romantic (Delibes and Herold), two Twentieth Century music (Khachaturian and Gould)	150	Same five conditions as in Radocy (1976), exp. 1. M1 and S1 contained information with positive bias toward the first presented stimulus for each music style, whereas M2 and S2 contained information with positive bias toward the second presented stimulus for each music style	Aggregate of 9 variables relating to quality, enjoyment, and interest	B	One-way ANOVA produced a significant main effect of condition. Specific *post hoc* results are not reported, although the authors state that only 11 of 36 *post hoc* comparisons by condition were significant
Silvey, 2009	Six excerpts (30 s) of a high school concert band	157	The participants were concert band students, wind ensemble students, or high school band directors and heard the series of six excerpts twice. A total of 80 participants received no accompanying information (control). Furthermore, 77 participants were informed that these were performances by two different bands, and each excerpt was labeled^c^ as either “concert band” or “wind ensemble”. The design was such that each excerpt was labeled once as each band type	Aggregate of tone; intonation; expression (interpreted as quality)	B	Mixed-design ANOVA produced a significant interaction of band label and presentation order for two pairs of excerpts (out of six pairs). For one pair, concert band students rated the excerpt higher when it was labeled as a concert band; for the other pair, concert band students rated the excerpt higher when it was labeled as a wind ensemble
Steinbeis and Koelsch, 2009	60 atonal excerpts by Webern and Schoenberg. Excerpts 8–13 s, containing at least one entire phrase	12	The excerpts were framed as either composed by a person or computer-generated, whereas in actuality all were created by renowned composers	Pleasantness	C	Least-squares estimation using the general linear model for serially autocorrelated observations produced no significant difference in pleasantness between the conditions
Ziv and Moran, 2006	Chopin’s Prelude in E minor, Op. 28, No. 4. Two versions were produced, with one at 55 bpm and the other at 62 bpm	96	A commercially available recording was converted to MIDI, and several duration and dynamic parameters were altered to make it sound “natural”. After two tempo variations were made, the excerpts were exported as audio files with reverb and compression. The participants heard both versions, with one version labeled as a performance by a pianist and the other as computer-generated	Preference (binary choice)	A	Repeated-measures MANOVA produced a significant main effect of label, with the human-labeled performance preferred; this was confirmed with a *post hoc* test. No significant differences were observed regarding presentation order

*Framing had to be manipulated in terms of associated quality (as described in the “Method” section for study 1). For cases where a study contains multiple experiments, each experiment is listed separately. The results are categorized as either A, B, or C. ^*a*^“Exp.” is the abbreviation used to refer to an experiment number within a study. ^*b*^“A” denotes results in which significant increases in preference and/or quality are observed alongside positively valenced framing. “B” denotes inconclusive results within the same experiment, such as both significant and non-significant results within the same experiment. “C” denotes results in which no significant increases in preference and/or quality are observed alongside positively valenced framing (see “Method” section for details). ^*c*^Silvey (2009, p. 48) noted that wind ensembles typically contain the most advanced students, and so this label was hypothesized to produce a positive bias.*

### Discussion

In contrast to Chmiel and Schubert’s (2019a) review, in which 21% of studies reported a significant increase in preference alongside additional historical framing, the present literature review on valenced framing reported a significant increase in preference for 50% of the examined experiments. Based on the data at hand—and counter to Bullot and Reber’s (2013b) PHF—we suggest that positive framing may have a greater positive impact on music than historical framing. However, no studies as yet have directly compared these types of framing within the same experimental design.

An additional finding reported by Chmiel and Schubert (2019a) was a discrepancy in the type of stimuli used between the two examined mediums (music works and visual works). The music stimuli used in the existing literature were almost exclusively of a “typical” nature, such as popular music and accessible forms of classical music and jazz (that is, not including styles such as atonal music or experimental/free jazz). Only one experiment (Bradley, 1972) contained music that could be considered unusual to many listeners (in this case, atonal music). In contrast, 50% of the experiments on visual art works exclusively examined abstract works, with an additional 32% examining both abstract and representational works. This difference in the types of investigated stimuli between studies on music works and visual works is noteworthy considering that a common hypothesis in the literature concerning visual works suggests that abstract stimuli should be more susceptible to any positive effects of framing in comparison to representational (typical) works (see, e.g., Temme, 1992, p. 29; Leder et al., 2006, p. 179; Bordens, 2010, p. 113; Specht, 2010, p. 194; Swami, 2013, p. 286). This hypothesis is based on an assumed increase in difficulty for understanding and interpreting an abstract work, which framing may ameliorate. Study 1 produced a comparable finding to Chmiel and Schubert’s review in terms of the music stimuli used. That is, only one experiment within study 1 [namely, that by Steinbeis and Koelsch (2009), which used atonal music by Schoenberg and Webern] contained music that could be considered as unusual. Steinbeis and Koelsch did not report any significant difference in pleasantness between conditions, although a relatively low sample size (*N* = 12) may account for this (VanVoorhis and Morgan, 2007).

Our review identified only one qualitative study (Bennett and Ginsborg, 2018) related to framing and music preference. Bennett and Ginsborg exposed participants to the same music twice, although framing was only present for the second exposure. This study was excluded from our literature review because the survey questions provided to the respondents did not explicitly refer to preference or an equivalent term but instead focused on whether or not they had listened to the music in “a different way once the background information was given” (p. 593). Thus, while 39% of the participants reported a “positive impact” of framing, we cannot conclude that framing did not have a positive impact for the remaining 61% of the participants as it was not included as an explicit part of the question. Regardless, there is a lack of available qualitative data on this topic. We call for further implementation of qualitative and mixed-methods approaches concerning framing and preference, which may identify key areas where framing has the most efficacy.

## Study 2 – Preference for Unusual Music With Combined Historical and Valenced Framing

Based on the above-mentioned findings, we conducted an empirical investigation on the impact of framing on preference for unusual music, which has been neglected in prior research. Furthermore, we decided to use a novel approach that incorporated both valenced framing *as well as* historical framing within the same provided text. This decision was made for two reasons: first, we surmised that the joining of two types of framing might produce a greater overall impact on preference than has been observed in the literature for each framing type individually (see study 1). Second, while a small amount of research (e.g., Swami, 2013) has explicitly tested the PHF, these designs have focused on historical framing alone rather than on valenced framing or a mix of the two. Indeed both types of framing can be explained by the PHF in broad terms of the variable “understanding”. The central hypothesis of the PHF suggests that if the respondents understand the historical details surrounding a work, this will enable the highest level of appreciation (preference) for that work. The PHF could be interpreted as predicting a positive relationship between preference and historical understanding, although one aspect of the PHF, known as the *esthetic–artistic confound* [see Bullot and Reber (2013a, 2017)], suggests that this impact on preference is not necessarily a positive one. As an example, Bullot and Reber (2017) discuss Leni Riefenstahl’s 1935 film *Triumph of the Will* in the context of the PHF and note that general reactions to the film moved from positive in the years before the Second World War to negative in the following years, presumably due to political ramifications of the film’s association with the Third Reich. While Bullot and Reber’s focus remains on historical understanding, we can also see a distinct difference in valenced framing for the film between the pre- and the post-war time periods, and as such, we interpret this as an example of combined historical and valenced framing. Such a combination of framing has not, to our knowledge, been examined empirically [although Rigg (1948) comes close].

Additionally, we noted that 11 of the 12 (92%) music experiments contained within Chmiel and Schubert (2019a) utilized a control that received no information at all, compared with a framed condition (these experiments were from Damon, 1933; Rigg, 1948; Bradley, 1972; Prince, 1974; Zalanowski, 1986; Halpern, 1992; Margulis, 2010; Vuoskoski and Eerola, 2015; Anglada-Tort et al., 2019). Based on the approach taken by Margulis et al. (2015), who used a “placebo program note” containing the architectural details of the performance venue to counterbalance the condition receiving a musical program note, we surmised that an absence of any information or enrichment whatsoever may produce a confounding, unintended effect. That is, the participants who receive framing may produce higher preference ratings than those receiving no information simply due to the presence of any enrichment, regardless of whether the enrichment was historical or non-historical, or whether the enrichment was positively/negatively framed or not. Consequently, we used an active control referred to as *imaginative engagement*, being a form of framing that we define as non-historical enrichment in which the participants were asked to freely form mental imagery while listening. This particular condition was not intentionally framed in either a positive or a negative manner.

Our decision to use imaginative engagement as an additional condition (instead of using a passive control condition) was based on the small amount of research on the possible impact of imagination on preference for music. Zalanowski (1986) investigated the influence of different ways of engaging with excerpts of classical music. One of the conditions requested the participants to form mental images while listening to the music, with no specific guidelines other than “try[ing] to develop these images as fully as possible” (p. 45; hence, a “free imagery” condition). Those in a second, “pay attention” condition were simply asked to listen to the music carefully. Additionally, three separate conditions contained program notes in some form. The first of these was a “structured imagery” condition, in which participants received compositional details alongside a description of the portrayed story, and were instructed to mentally form the images suggested by this story. A second program condition received “abstract” program notes that linked the mood of the work to specific instruments and sections, whereas a third program condition received “analytical” program notes containing technical details of the music and suggested sections to listen for.

While one stimulus in Zalanowski’s study produced no significant impact of condition, for the other stimulus the free imagery condition produced the highest level of enjoyment of the conditions investigated. In contrast, the structured imagery condition was not associated with enjoyment that was greater than the other conditions. Based on this study, free imagery could impact positively on preference but structured imagery might not. We decided to examine the influence of imagination directly in comparison to historical framing to explore the influence it might have in this context. Imagery also has an advantage over passive control (no explicit instruction to engage with the music in any particular way) because it would bring those listeners into a more homogenous way of engaging with the music. A passive control will leave listeners free to do what they wish, possibly leading to a wide variety of (uncontrolled) ways of engaging and (on the assumption that self-framing/engagement impacts on preference) leading to a wide range of preference scores. The PHF makes no explicit prediction about how imaginative engagement would impact on music except that, since such engagement does not necessarily lead to improved understanding (in comparison to a historically relevant task), our conception of the PHF predicts that historical framing will produce higher preference scores than imaginative engagement will.

Study 2 therefore tests two hypotheses, each inspired by Bullot and Reber’s (2013b) PHF as well as the findings of study 1:

(H1) Preference ratings for an unusual piece will be higher when accompanied by positive historical framing than when accompanied by negative historical framing.
(H2) Preference ratings for an unusual piece will be higher when accompanied by positive historical framing than when accompanied by non-historical (imaginative) enrichment.

We also collected additional data (complexity, familiarity, puzzlingness, and interest, henceforth “secondary variables”). While these variables are known to be related to preference (e.g., Berlyne, 1960, 1971, 1974; Martindale and Moore, 1989; Martindale et al., 1990; North and Hargreaves, 2000; Silvia, 2005; Hargreaves and North, 2010; Chmiel and Schubert, 2017), our intention for including the secondary variables was to help us to ascertain the unusualness (characterized by high complexity, high puzzlingness, and low familiarity) of the stimuli selected and to help explain any unexpected results.

### Method

#### Materials

A set of five pieces of music was used, as listed in Table 2. These consisted of one piece of interest (an “unusual test piece”) that was used to manipulate framing and four “other pieces” that acted to create a program of varying music without drawing attention to the specific interest that we have in the test piece. Each piece consisted of an excerpt, approximately 2 to 3 min in duration, taken from a larger work. The test piece was in fact two short pieces taken from Webern’s “Six Pieces for Orchestra, Op. 6” joined together as one continuous excerpt (see Table 2 for details). This excerpt is henceforth referred to as *Six Pieces for Orchestra*. Being atonal music, this piece was intended as an example of music that the general public might consider unusual or even extreme. The remaining pieces consisted of a jazz rendition and improvisation based on the melody and the chords of a rock song (*Black Hole Sun*), a well-known pop song (*Bohemian Rhapsody*), a world music excerpt (*Kora Demonstration*), and a pastiche composition designed from the results of a survey asking what musical and lyrical elements people found most undesirable (*The Most Unwanted Song*).

**TABLE 2:** Details of pieces used in study 2.

Piece label	Piece function	Piece details	Excerpt duration
*Six Pieces for Orchestra*	Unusual test piece	Webern, A. (1909). “Six Pieces for Orchestra, Op. 6”, On *Schoenberg, Webern, Berg: Orchestral Works* [CD]. London: Warner Classics, 2003	2.28
*Black Hole Sun*	Other piece	Cornell, C. (1994). “Black hole sun” [performed by Brad Mehldau Trio], on *Brad Mehldau Trio Live* [CD]. New York: Nonesuch Records, 2008	3:23
*Bohemian Rhapsody*	Other piece	Mercury, F. (1975). “Bohemian rhapsody”, on *A Night at the Opera* [CD]. London: EMI Records	3:10
*Kora Demonstration*	Other piece	Diabate, T. (2008) “Kora demonstration at WOMAD 2008” [performed by Toumani Diabate and the Symmetric Orchestra], live recording accessed from *YouTube*	2:33
*The Most Unwanted Song*	Other piece	Soldier, D. (1997). “The most unwanted song”, on *The People’s Choice: Music* [CD]. New York: Mulatta Records	2:48

*The unusual test piece is listed first, although stimulus presentation to participants was randomized across classes. The first two pieces from *Six Pieces for Orchestra* were combined into one continuous excerpt, which contained both pieces in their entirety with approximately 5-s gap in between.*

#### Participants

One hundred and eighteen participants were recruited from an Australian undergraduate elective course containing a mixture of music students and non-music students. The participants were asked how many years they had spent playing a musical instrument/singing and also how many years they had received any form of training on any musical instrument/voice. The participants who responded with 6 or more years for either question were categorized as “trained”, whereas those who responded with 5 years or less to both questions were categorized as “novice”, based on guidelines proposed by Zhang and Schubert (2019). This variable is henceforth referred to as *musicianship*. The sample contained 65 females (55%) and 53 males (45%), with age ranging from 18 to 28 years (*M* = 20.6, SD = 1.9). There were 58 novices (49%) and 60 trained participants (51%), with “years playing” ranging from 0 to 20 years (*M* = 5.3, SD = 5.2) and “years training” ranging from 0 to 16 years (*M* = 5.3, SD = 5.2).

#### Procedure

The participants were tested in their existing undergraduate classes. There were three classes, each containing between 30 and 46 people, with a separate testing room used for each class. The participants made their responses with a personal device (laptop or tablet) or were provided with a computer. First, the participants provided their demographic information using an online survey created in *Key Survey*. Following this, they listened to their selection of five music excerpts over a common loudspeaker. Each excerpt in the set was played once, and stimulus ordering was randomized across classes. Before each excerpt was played, a vignette that provided instructions for framing or engagement was displayed on a large screen that all participants in the class could see, and the vignette was read out by the instructor. The vignette remained on the screen during listening and for approximately 2 min afterward. The verbatim text for each vignette is provided in the Supplementary Material.

For the test piece, the three classes each received a different vignette that contained either (1) *positive historical framing, (2) negative historical framing, or (3) imaginative engagement.* Thus, for the test piece, three conditions were created, hence referred to as the positive, negative, and imaginative conditions. For each of the four other pieces, the participants in all classes received an identical, brief historical vignette that is supplied in the Supplementary Material. The participants in the positive and the negative conditions received information on the compositional and stylistic aspects of the music, suggested elements to listen for, and the title, composer, and year of publication/release. Additionally, each positive condition was positively framed through suggestion that aspects of the music were groundbreaking or revolutionary and well received, whereas each negative condition was negatively framed by suggesting that the piece was not well received. Each imaginative condition asked the participants to freely form mental imagery while listening. Thirty participants received the positive condition, 46 participants received the negative condition, and 42 participants received the imaginative condition.

The participants used the online survey to provide responses for the variables preference, complexity, familiarity, puzzlingness, and interest. Each variable was recorded on 11-point scales (0–10), with the scales labeled “I like this piece”, “The music sounds complex”, “This piece is highly familiar”, “The music is puzzling”, and “The music is interesting”. The participants used the response guides “strongly agree (10)”, “strongly disagree (0)”, and “neither agree nor disagree” (5) for all variables. The participants were allowed to record their responses either while the excerpt was playing or during the 2 min after each excerpt had finished playing. As the primary focus of this study was to examine the preference ratings for the different conditions, all preference ratings were set as mandatory for the submission of the survey. The four secondary variables were used to validate the selection of the test piece. These variables were not mandatory to minimize the time the participants in a class had to wait before continuing with the next example, and completed responses were used to estimate the overall level of the secondary variables. Regardless, the participants were instructed to respond to all of the five rating scales.

### Results

**FIGURE 1:**
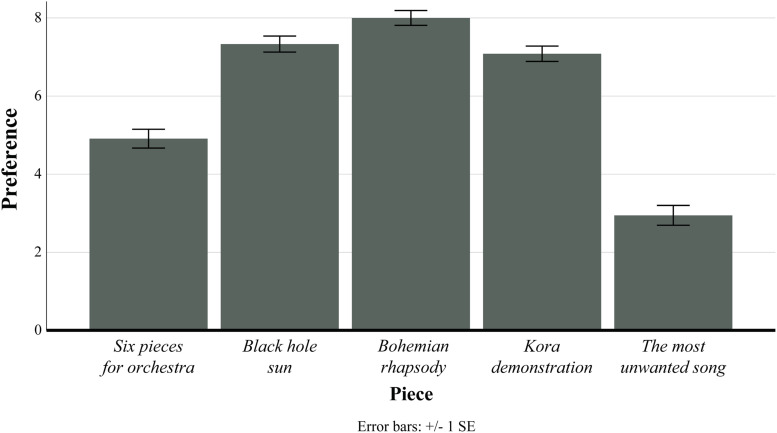
Plot of mean and error bars of preference ratings for each piece used in study 2. For this analysis, all three conditions for *Six Pieces for Orchestra* were collapsed.

As preference ratings were mandatory for the submission of the survey, this resulted in 590 data entries for this variable. Ratings of the secondary variables were not mandatory items; of the 2,360 maximum possible ratings of these four combined variables, 2,259 (96%) were recorded. We conducted our analysis of the sample without omission of the participants who did not supply ratings of these variables. To check that the test piece was unusual, we first inspected the preference ratings between the five pieces, collapsing the three conditions for *Six Pieces for Orchestra*. The descriptive statistics of preference for each piece are reported in Table 3 and are plotted in Figure 1. A within-subjects ANOVA containing preference as the dependent variable and piece as the independent variable produced significant results [*F*(4, 468) = 101.44, *p* < 0.001, η*_*p*_^2^* = 0.484]. Bonferroni-corrected *post hoc* tests (see Supplementary Table 1) indicated that *Six Pieces for Orchestra* and *The Most Unwanted Song* were rated significantly lower than all other stimuli. Furthermore, complexity was rated highest, puzzlingness was rated equal highest, and familiarity was rated lowest for *Six Pieces for Orchestra* in comparison to the other excerpts, together confirming that it was the most unusual stimulus in the set (the descriptive statistics for all secondary variables are also reported for each piece in Table 3).

**TABLE 3:** Descriptive statistics of preference and the four secondary variables from study 2.

Piece	Statistic	Preference	Complexity	Familiarity	Puzzlingness	Interest
*Six Pieces for Orchestra*	*M*	4.83	7.08	2.79	5.88	6.31
	SD	2.54	2.29	2.72	2.85	2.65
	*n*	118	117	115	102	117
*Black Hole Sun*	*M*	7.35	5.25	4.91	2.53	6.95
	SD	2.12	2.31	2.98	2.45	2.18
	*n*	118	115	115	112	114
*Kora Demonstration*	*M*	7.04	7.17	6.02	3.52	7.36
	SD	2.06	2.06	3.11	2.61	2.10
	*n*	118	117	116	110	118
*Bohemian Rhapsody*	*M*	8.03	6.39	7.91	3.33	7.92
	SD	1.95	2.16	2.81	2.51	1.95
	*n*	118	111	116	107	114
*The Most Unwanted Song*	*M*	2.93	5.22	4.69	5.88	4.91
	SD	2.63	2.26	3.42	2.65	3.05
	*n*	118	111	113	108	111

*For the variable preference, the three framing conditions for *Six Pieces for Orchestra* have been collapsed. The rating scales for all variables contained the labels “strongly agree (10)”, “strongly disagree (0)”, and “neither agree nor disagree (5)”.*

**FIGURE 2:**
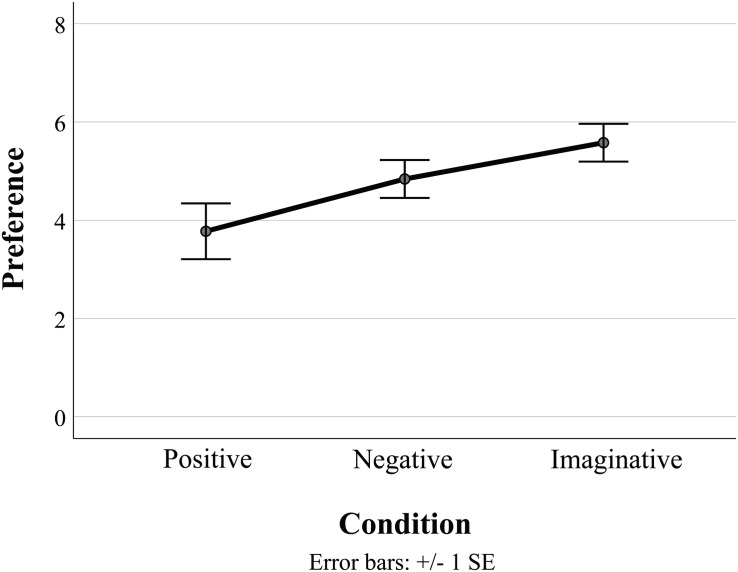
Plot of mean and error bars of preference ratings for the test piece *Six Pieces for Orchestra* (study 2), split by condition. The only significant difference observed was between the positive and the imaginative conditions (*p* = 0.029, *d* = 0.548).

*Six Pieces for Orchestra* was then subjected to further analysis, with a specific focus on comparing the preference ratings for the three conditions, in addition to sex and musicianship as independent variables. The descriptive statistics of preference for *Six Pieces for Orchestra* are shown across the three conditions in Table 4 and plotted in Figure 2. A Shapiro–Wilk test indicated that each condition of the piece was normally distributed (*p* > 0.05), and a Levene test indicated homogeneity of variances (*p* = 0.296). No significant three-way interaction of condition × musicianship × sex was observed [*F*(2, 106) = 2.01, *p* = 0.139, η*_*p*_^2^* = 0.037], and no significant two-way interactions were observed for condition × musicianship [*F*(2, 106) = 1.60, *p* = 0.206, η*_*p*_^2^* = 0.029] or condition × sex [*F*(2, 106) = 0.56, *p* = 0.574, η*_*p*_^2^* = 0.010]. A significant main effect was observed for condition [*F*(2, 106) = 3.52, *p* = 0.033, η*_*p*_^2^* = 0.062], although no significant main effects were observed for musicianship [*F*(1, 106) = 0.70, *p* = 0.405, η*_*p*_^2^* = 0.007] or sex [*F*(1, 106) = 2.04, *p* = 0.156, η*_*p*_^2^* = 0.019]. Thus, all subsequent analyses for this piece only examine the independent variable condition. Bonferroni-corrected *post hoc* tests examining the preference ratings between conditions produced no significant difference between the positive and the negative conditions (*p* = 0.372, *d* = 0.169), although participants from the imaginative condition rated preference significantly higher than those in the positive condition (*p* = 0.029, *d* = 0.548). There was also no significant difference in preference between the negative and the imaginative conditions for *Six Pieces for Orchestra*, although a medium effect size was observed (*p* = 0.534, *d* = 0.425), according to Cohen’s (1992) guidelines.

**TABLE 4:** Descriptive statistics of preference ratings for the unusual test piece *Six Pieces for Orchestra* used in study 2, split by condition.

Framing condition	*M*	*SD*	*n*
Positive	3.78	2.56	30
Negative	4.84	2.65	46
Imaginative	5.58	2.26	42

### Discussion

In study 2, we examined whether positive *versus* negative historical framing would increase the preference ratings for a piece of unusual (atonal) music. First, the preference ratings were compared for participants receiving historical framing that was positively valenced (positive condition) and those receiving historical framing that was negatively valenced (negative condition), with the hypothesis that the positive condition would produce higher ratings (H1). Second, the ratings were compared for those receiving the positive condition and those receiving a non-historical, non-valenced enrichment (imaginative condition), again with the hypothesis that the positive historical framing would produce higher ratings (H2). The findings were counter to both of the hypotheses. Regarding H1, no significant difference was observed between the positive and the negative conditions, and this is supported by a small accompanying effect size. Regarding H2, we noted an unexpected result in which the imaginatively framed music was more preferred than the positive historically framed music, and this difference was accompanied by a medium effect size. The mean preference ratings were also higher for the imaginative condition compared to the negative condition. This difference did not reach significance, although it was accompanied by a medium effect size.

The observed positive impact of imagination is similar to the results reported by Zalanowski^3^ (1986). Based on H1 and H2, we expected historical framing—in particular, positive valenced framing—to produce increased preference. The equivocal and unexpected results of study 2 urged us to conduct a follow-up study to further examine the impact of imagination in comparison to historically valenced framing. Although we found evidence that the test stimulus was unusual, it may not have fulfilled the criterion of “extreme”, as discussed in study 1. This was particularly noticeable for the secondary variable puzzlingness. While *Six Pieces for Orchestra* received the equal highest mean rating for puzzlingness among the examined pieces in study 2 (alongside *The Most Unwanted Song*), this variable was only rated moderately (*M* = 5.88, SD = 2.85). Furthermore, in study 2, we observed the effect of framing on a low-preference stimulus (*Six Pieces for Orchestra*). Presumably, one reason why the preference for this stimulus may have been rated low is because it was unusual. Through manipulation of collative variables (familiarity, in particular), it is possible to induce low preference for music that is not unusual, for example, through over-exposure (see Berlyne, 1971; Finnäs, 1989; Chmiel and Schubert, 2017). Thus, in the following study, we aim to include an additional low-preference test stimulus for comparison, although of a typical nature.

## Study 3 – Comparing Framing for Unusual Versus Typical, Over-Familiar Music

Study 3 expands study 2 in three ways. First, as we could identify only two prior studies containing framing and imagination [being study 2 and Zalanowski (1986)], we examine whether the positive impact of imagination on preference can be reproduced. Regardless of the positive impact of imagination observed in study 2, we retained H2, according to which the imaginative condition will produce lower ratings of preference than the positive condition. Second, study 3 aims to examine framing for a piece of music that was specifically chosen to be more unusual than the atonal piece used in study 2, which received only moderate ratings of puzzlingness. Third, study 3 manipulates framing for an unusual test piece as well as a typical, over-familiar test piece. Due to the inclusion of a typical stimulus for comparison, a third hypothesis (H3) was added to H1 and H2. H3 was drawn from literature concerning framing for visual works (see “Discussion” section in study 1):

(H3) Any positive impact of historical, valenced framing on preference will be enhanced for *a priori* low-preference unusual music in comparison to *a priori* low-preference typical music.

### Method

#### Materials

A set of five pieces of music was used, as listed in Table 5. Framing was only manipulated for the unusual test piece and the typical test piece. The unusual test piece (*Elevation*) was an excerpt of Free jazz by Pharoah Sanders. Free jazz was specifically chosen with the intention of being more unusual (i.e., stylistically unfamiliar, complex, and puzzling) than the atonal excerpt used in study 2, partly based on its use in a prior study (Hargreaves, 1984). The typical test piece was a recording of the piano composition *Allegro Burlesque* by Friedrich Kuhlau. As with study 2, the remaining three “other pieces” acted to create a program of varying music without drawing attention to the specific interest we have in the test pieces. The other pieces consisted of two popular works from different decades (*Ain’t No Mountain High Enough* and *Thrift Shop*) and a piece taken from Beijing Opera (*The Drunken Concubine*). Apart from *Allegro Burlesque* (which consisted of the piece in its entirety), each piece consisted of an excerpt approximately 2 to 3 min in duration, taken from a larger work.

**TABLE 5:** Details of pieces used in study 3.

Piece label	Piece function	Piece details	Excerpt duration
*Elevation*	Unusual test piece	Sanders, P. (1973). “Elevation”, on *Elevation* [CD]. Los Angeles: Impulse	3:14
*Allegro Burlesque*	Typical test piece	Kuhlau, F. (1827). “Allegro burlesque” from Four Sonatinas, Op. 88 (see main text for details on the production of this piece)	1:49
*Ain’t no Mountain High Enough*	Other piece	Ashford, N. and Simpson, V. (1966). “Ain’t no mountain high enough” [performed by Marvin Gaye and Tammi Terrell]. On *United* [CD]. Detroit: Tamla Records, 1967	2:27
*The Drunken Concubine*	Other piece	Lanfang, M. (1930). “The drunken concubine”, on *Famous Arias from Peking Opera vol. 1* [CD]. Beijing: China Record Corporation, 2006	2:38
*Thrift Shop*	Other piece	Haggerty, B. (aka Macklemore) and Lewis, R. (2012). “Thrift shop”, on *The Heist* [CD]. Seattle: Macklemore LLC, 2012	2:25

*The unusual test piece is listed first and the typical test piece is listed second, although stimulus presentation to participants was randomized across classes. *Elevation* commenced with a fade in, starting at 4 min and 40 s into the piece, whereas all other excerpts commenced at the opening of the piece.*

#### Participants

One hundred and five participants were recruited from the same Australian undergraduate elective course as study 2, although no participants from study 2 were used in this sample. As before, the sample contained a mixture of music students and non-music students, and the participants were categorized as either “trained” or “novice” using the same method as in study 2. The sample contained 60 females (57%) and 45 males (43%), with age ranging from 18 to 32 years (*M* = 20.8, SD = 2.3). There were 54 novices (51%) and 51 trained participants (49%), with “years playing” ranging from 0 to 20 years (*M* = 6.1, SD = 5.6) and “years training” ranging from 0 to 16 years (*M* = 4.3, SD = 4.7).

#### Procedure

Data collection occurred several weeks after that for study 2. The participants were tested in their existing undergraduate classes. There were three classes, each containing between 28 and 42 people, with a separate testing room used for each class. Data collection was identical to study 2, with responses made on an online survey created in *Key Survey*. Each excerpt in the set was played once in a different randomized order across each class over a common loudspeaker, and before each excerpt was played, a vignette that provided framing was read out by the instructor and also displayed on a large screen such that all participants in the class could see. The vignette remained on the screen during listening and for approximately 2 min afterward. The verbatim text for each vignette is supplied in the Supplementary Material. In the fortnight leading up to study 3, all participants from this sample took part in an unrelated study (Canazza et al., 2013; Schubert et al., 2014a, 2017) in which they were exposed to seven different versions of *Allegro Burlesque* (six computer-generated versions and also the human-performed version that is used in this study), which had the effect of making them highly familiar (and possibly “over-familiar”) with the piece. In the earlier study, the participants listened to each version at least once, although there was no limit to the number of times that they could replay each version. Thus, we expected that the participants would be highly familiar with *Allegro Burlesque*, producing a further distinction between this as a typical, familiar piece and the unusual, unfamiliar test piece (*Elevation*).

As with study 2, for the two test pieces the three classes each received a different vignette that contained either (1) positive historical framing, (2) negative historical framing, or (3) imaginative engagement. The participants did not receive the same condition for both of their test pieces. For each of the three other pieces, the participants in all classes received an identical, brief historical vignette that is supplied in the Supplementary Material. The participants used the online survey to provide responses for the same five variables (preference, complexity, familiarity, puzzlingness, and interest), with each recorded on 11-point scales (0–10) and labeled as per study 2. The participants were allowed to record their responses either while the excerpt was playing or during the 2 min after each excerpt had finished playing. As before, all preference ratings were set as mandatory for the submission of the survey, whereas the four secondary variables were not set as mandatory for the submission of the survey. The participants were again instructed to respond to all of the five rating scales. Thirty-five participants received the positive condition for *Elevation* and also the negative condition for *Allegro Burlesque*, 42 participants received the negative condition for *Elevation* and also the imaginative condition for *Allegro Burlesque*, and 28 participants received the imaginative condition for *Elevation* and also the positive condition for *Allegro Burlesque*.

### Results

**FIGURE 3:**
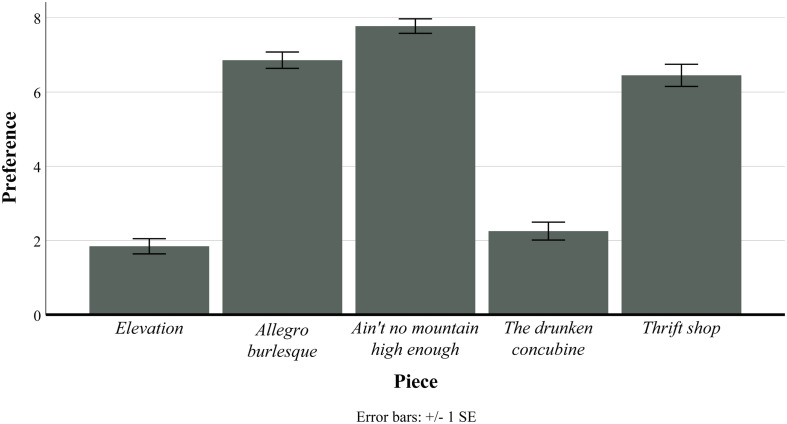
Plot of mean and error bars of preference ratings for each piece used in study 3. For this analysis, all three conditions of *Elevation* were collapsed, and all three conditions of *Allegro burlesque* were collapsed.

As preference ratings were mandatory for the submission of the survey, this resulted in 525 data entries for this variable. Ratings of the secondary variables were not mandatory items; of the 2,100 maximum possible ratings of these four combined variables, 2,039 (97%) were recorded. We conducted our analysis of the sample without omission of the participants who did not supply ratings of these variables. The preference ratings for the five stimuli collapsed across conditions were inspected. The descriptive statistics of preference for each piece are reported in Table 6 and plotted in Figure 3. A within-subject ANOVA containing preference as the dependent variable and piece as the independent variable produced significant results [*F*(4, 416) = 150.13, *p* < 0.001, η*_*p*_^2^* = 0.607]. The Bonferroni-corrected *post hoc* tests (see Supplementary Table 2) indicated that *Elevation* was rated significantly lower in preference than all pieces apart from *The Drunken Concubine*. *Allegro Burlesque* was rated significantly higher than *Elevation* and *The Drunken Concubine* and significantly lower than *Ain’t No Mountain High Enough*, whereas this piece was not rated significantly different in preference to *Thrift Shop*. These data also reveal that the preference ratings for *Allegro Burlesque* were relatively high (*M* = 6.79, SD = 2.19), counter to our predictions based on the impact of prior exposures. In addition, *Elevation* had the highest complexity and puzzlingness ratings and also the lowest familiarity rating of the stimuli, whereas *Allegro Burlesque* had an overall high familiarity rating and a low puzzlingness rating.

**TABLE 6:** Descriptive statistics of preference and the four secondary variables from study 3.

Piece	Statistic	Preference	Complexity	Familiarity	Puzzlingness	Interest
*Elevation*	*M*	1.93	5.82	0.67	6.81	4.29
	SD	2.07	3.05	1.20	3.15	3.23
	*n*	105	104	103	103	105
*Allegro Burlesque*	*M*	6.79	6.13	7.79	2.83	6.78
	SD	2.19	2.04	2.72	2.33	1.91
	*n*	105	104	104	91	104
*Ain’t No Mountain*	*M*	7.75	4.27	8.21	1.72	6.97
	SD	1.93	1.96	2.44	1.87	2.05
	*n*	105	105	105	100	105
*The Drunken Concubine*	*M*	2.28	4.94	6.45	5.72	4.01
	SD	2.40	2.66	3.21	2.92	2.90
	*n*	105	103	101	97	102
*Thrift Shop*	*M*	6.37	4.23	7.88	2.38	6.22
	SD	2.94	2.28	3.15	2.49	2.64
	*n*	105	102	102	96	102

*For the variable preference, the three framing conditions for *Elevation* have been collapsed, and the three framing conditions for *Allegro Burlesque* have also been collapsed. The rating scales for all variables contained the labels “strongly agree (10)”, “strongly disagree (0)”, “neither agree nor disagree” (5).*

**FIGURE 4:**
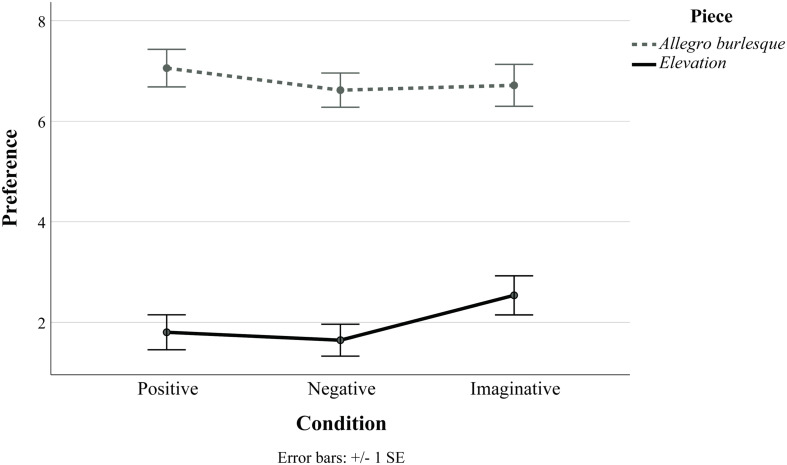
Plot of mean and error bars of preference ratings for the two test pieces in study 3, split by condition. Separate non-parametric tests on each piece produced no significant differences in preference between conditions for either piece.

*Elevation* and *Allegro Burlesque* were then examined, with a specific focus on comparing the preference ratings for the three framing conditions. The descriptive statistics of preference for *Elevation* and *Allegro Burlesque*, split across the three conditions, are reported in Table 7 and also plotted in Figure 4. Before the analysis of preference ratings, the normality of data for each condition of each test piece was assessed with a Shapiro–Wilk test. Each condition of *Elevation* was atypically distributed (positive condition *p* < 0.001; negative condition *p* < 0.001; imaginative condition *p* = 0.003), and two of the three conditions for *Allegro Burlesque* were atypically distributed (positive condition *p* = 0.011; negative condition *p* = 0.275; imaginative condition *p* = 0.006). Due to the violation of the normality assumption, separate non-parametric tests were conducted for each of the two pieces instead of an ANOVA.

**TABLE 7:** Descriptive statistics of preference for the two test pieces from study 3 (*Elevation* and *Allegro Burlesque*).

Piece	Framing condition	*M*	SD	*n*
*Elevation*	Positive	1.80	2.07	35
	Negative	1.64	1.90	42
	Imaginative	2.54	2.27	28
*Allegro Burlesque*	Positive	7.06	2.10	28
	Negative	6.62	2.09	35
	Imaginative	6.71	2.49	42
Both test pieces collapsed	Positive	4.16	3.38	63
	Negative	3.86	3.03	77
	Imaginative	5.10	3.23	70

*These data are first reported split by condition and piece and next reported collapsed over the two pieces, but split by condition.*

For *Elevation*, a non-parametric Levene test (see Nordstokke and Zumbo, 2010) was used to examine the pooled rank scores; these data did not violate the non-parametric homogeneity of variance assumption (*p* = 0.291). Following this, a Kruskal–Wallis *H* test was used to examine differences in preference ratings by condition (which contained three levels). The median preference ratings were 1.0 for the positive condition, 1.0 for the negative condition, and 2.0 for the imaginative condition, although no significant differences were observed between the conditions [*H*(2) = 2.90, *p* = 0.235]. For the variables musicianship and sex, which each contained two levels, a separate Mann–Whitney *U* test was conducted for each independent variable. The median preference ratings for novices (1.0) and trained participants (2.0) were not statistically different (*U* = 1498.5, *z* = 0.80, *p* = 0.422), although the median preference ratings were statistically (*U* = 1651.5, *z* = 2.03, *p* = 0.042) higher for males (2.0) than for females (1.0). The *M* (SD) preference ratings for this piece were 2.42 (2.25) for males and 1.57 (1.86) for females and 1.77 (2.02) for novices and 2.10 (2.13) for trained participants.

For *Allegro Burlesque*, a non-parametric Levene test confirmed that the pooled rank scores did not violate the homogeneity of variance assumption (*p* = 0.294). As mentioned above, a Kruskal–Wallis *H* test was used to examine differences in preference ratings by condition. The median preference ratings were 7.0 for the positive condition, 7.0 for the negative condition, and 7.5 for the imaginative condition; no significant differences were observed between conditions [*H*(2) = 2.36, *p* = 0.308]. Separate Mann–Whitney *U* tests were conducted for the independent variables musicianship and sex. The median preference ratings for novices (7.0) and trained participants (7.0) were not statistically different (*U* = 1566.5, *z* = 1.23, *p* = 0.220). Similarly, the median preference ratings for male (7.0) and female participants (7.0) were not statistically different (*U* = 1179, *z* = −1.12, *p* = 0.263). The *M* (SD) preference ratings for this piece were 6.53 (2.14) for males and 6.98 (2.24) for females and 6.50 (2.38) for novices and 7.10 (1.96) for trained participants. Based on these analyses, we concluded that valenced historical framing did not have a greater positive impact on the unusual piece than for the typical piece (rejecting H3).

**FIGURE 5:**
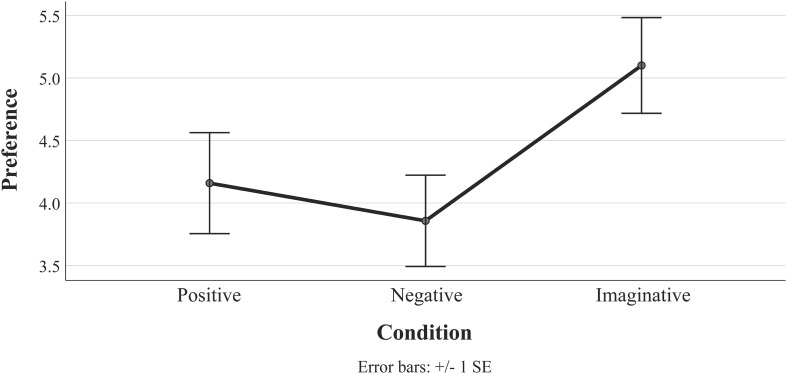
Plot of mean and error bars of preference ratings for the two test pieces in study 3 (*Allegro burlesque* and *Elevation)*, which have been collapsed across both pieces and split by condition in an *ad hoc* analysis.

The median^4^ ratings reported above as well as the error bars in Figure 4 for *Elevation* suggest that the imaginative condition might again produce a positive impact on preference. Based on the visual similarity of the imaginative condition plots for *Elevation* (study 3, in Figure 4) and *Six Pieces for Orchestra* (study 2, in Figure 2), we decided to run an additional analysis. As each participant in study 3 received more than one type of framing (split between the two test stimuli), it was not possible to directly compare the impact of framing between the two test pieces (e.g., with a 2 × 3-design ANOVA containing piece and condition as independent variables). However, a one-way ANOVA was performed with the preference ratings for both test pieces in study 3 collapsed and the three conditions used as an independent variable. The one-way ANOVA is considered robust against deviations against normality when the sample sizes are not small (Sawilowsky and Blair, 1992; Blanca et al., 2017; Maxwell et al., 2017), which was the case for the collapsed data (each condition ranged in size from 63 to 77 responses). Regardless, caution should be taken with the interpretation of these data, which are reported in Figure 5 and Table 7. A Levene test indicated homogeneity of variances (*p* = 0.253), and a main effect of condition was marginally non-significant [*F*(2, 207) = 2.94, *p* = 0.055, η*_*p*_^2^* = 0.028]. Games–Howell *post hoc* tests were used due to the non-normally distributed data. When collapsed across the two test pieces, the imaginative condition produced significantly higher ratings of preference than the negative condition (*p* = 0.046, *d* = 0.396), which is supported by a medium effect size. No significant differences were observed between the positive and the negative conditions (*p* = 0.847, *d* = 0.093) or the positive and the imaginative conditions (*p* = 0.233, *d* = 0.284), although this latter comparison was again accompanied by a medium effect size.

### Discussion

As with study 2, the results produced in study 3 were counter to all three hypotheses. First, there were no significant differences in preference between the historical conditions for either test piece (rejecting H1). While no significant effect of condition was observed for *Elevation* or for *Allegro Burlesque*, when we examined the median preference ratings for the conditions as well as the error bars of the mean scores, the data suggested a similar trend to study 2 in which the imaginative condition received slightly higher ratings (rejecting H2). An *ad hoc* analysis produced significantly higher preference ratings for the participants in the imaginative engagement condition than the positive historical framing condition, supported by a medium effect size. Based on this, we surmise that imaginative engagement seems to have a positive effect on music that is otherwise low in preference, although it is not yet clear if this is related to the unusualness of the music or how reproducible this effect is.

Second, no differences in preference were observed for the two historical conditions between the typical and the unusual pieces (rejecting H3). One interpretation is that unusual music is not impacted by historical framing differently to typical music (largely in line with the results of prior research on historical framing for strictly typical music, e.g., Prince, 1974; Margulis, 2010; Margulis et al., 2015), although due to the lack of additional research on this topic, we also cannot rule out the possibility that the music used in study 3 was still not unusual enough for this to occur. While we intended *Elevation* to be rated as highly unusual or even at extremely high levels of complexity and puzzlingness, these variables were rated moderately on a scale of 0 to 10 (complexity*M* = 5.82, SD = 3.05; puzzlingness *M* = 6.81, SD = 3.15). This is highlighted by the fact that *Elevation* was not rated significantly higher in complexity than *Allegro Burlesque*. Based on this, the next steps for future research on framing might benefit from the initial examination of H3 for non-musicians only, who are generally regarded as holding a lower tolerance for musical complexity and unconventionality (Orr and Ohlsson, 2005).

We must also acknowledge that *Elevation* and *Allegro Burlesque* differed not only in unusualness but also significantly in familiarity due to prior exposures to *Allegro Burlesque.* This prevents us from teasing apart whether the enrichment intervention benefited the preference ratings due to familiarity or due to unusualness. Future research on the impact of framing between typical and unusual music should aim to match stimuli in terms of familiarity as mere exposure has been shown to have a substantial impact on preference for music (Berlyne, 1971; Finnäs, 1989; Szpunar et al., 2004; Schellenberg et al., 2008; Margulis, 2014; Chmiel and Schubert, 2017). Furthermore, it is possible that framing and enrichment are highly subjective variables in that they impact people in different ways and are of little consequence to some listeners. Such a conclusion is supported by the heterogenous results observed in the existing literature and emphasizes the need for future work to include novel methods and the further integration of qualitative and mixed-method designs.

## General Discussion and Conclusion

This paper reported the results of a literature review on the impact of valenced framing on music preference (study 1) and two subsequent empirical studies on music preference for participants receiving one of three types of framing/enrichment (study 2 and study 3). In contrast to the findings of an existing literature review on the impact of historical framing (Chmiel and Schubert, 2019a), study 1 concluded that positively valenced framing had a significant, positive impact on preference 50% of the time. Additionally, study 1 further confirmed previous observations that existing studies on framing have almost exclusively contained typical music stimuli such as popular music and accessible forms of classical and jazz music. In study 2, we found no evidence of benefit to unusual music preference delivered by historical framing, whether positive or negative, although we did find that imaginative engagement increased the preference for unusual music. This finding was replicated in a follow-up study (study 3), where we went to greater lengths to select unusual music and also included a piece that was highly familiar and typical. The latter study replicated the increased preference for the imaginative condition as well as no difference between the two historical conditions, albeit with some statistical limitations.

The above findings prompt a rethink of our initial aim which was to test the PHF for unusual music. While the central hypothesis of the PHF proposes that an understanding of music should lead to greater preference for that music, we failed to empirically produce this outcome. In the following paragraphs, we will attempt to explain why and look for other ways forward. First of all, the PHF may still be valid but was not tested properly. In terms of our design for studies 2 and 3 and the bulk of research reviewed in study 1, the task that we present is somewhat artificial because it assumes that the listener will be able to absorb sufficient knowledge from a single exposure to information about the piece. It is possible that repeated pairings of the framing condition would provide greater opportunity for the information to be processed and connected with the music. However, even with a more thorough way to test the PHF, the improvements in preference associated with imaginative engagement require an alternative explanation.

We concluded from both study 2 and study 3 that imaginative engagement seems to have a significant, positive impact on preference for unusual music. The PHF makes no explicit prediction about the role of imaginative engagement upon preference. One explanation is that the repeated pairings of framing vignettes would also improve cognitive fluency, in line with the predictions by Belke and colleagues (e.g., Belke et al., 2010). Alternatively, these results fit the dynamic minimalist account proposed by Schubert et al. (2014b), which proposes that each of the cognitive factors that determine musical response also generate musical pleasure. In other words, any kind of mental representation associated with music can add to cognitive activation, which, they claim, generates pleasure. According to this view, pleasure can be generated by historical framing as much as imaginative engagement. This account provides a more compelling explanation of the results. Whether or not additional engagement with historical framing improves preference, the apparently immediate effect of imaginative engagement, as was also found by Zalanowski (1986), suggests that such engagement is immediately enriching for the listener. Historical framing may also be enriching but will take more time for the typical listener and may also be simply unappealing for some, leading them to avoid seeking out the formation of such connections. It may also be that the freedom associated with imaginative engagement simply allowed the participants the ability to recruit a much vaster array of mental representations with which the music could be linked during the listening experience, producing an overall greater amount of net mental activation [for further discussion, see Schubert (2013)].

This study has several limitations that need to be addressed in future research. First, we were only moderately successful in identifying stimuli that could be considered unusual. Perhaps our stimuli were not considered unusual or extreme because approximately half of the sample consisted of musically experienced people. While our tests indicated that musical background did not influence the preference results, future research may still find it easier to have pieces rated as unusual or extreme if the participants have generally low levels of musical experience. The recommendation to take this course of action is indicative of the nascent state of research on the relationship between framing/enrichment and preference. Second, the nature of the vignettes could be tested empirically beyond the construct validity produced by the research team. A separate study that ensures that the vignettes are clearly positive or negative in the qualities that they imbue upon the stimulus could be run. In the case of the designs applied to the present study, this would help to ensure that the positive and the negative vignettes were similar to the extent possible in every respect except for valence. Third, as a result of our attempt to make the empirical studies ecologically plausible, by having groups of listeners in a concert-like experience, we sacrificed much of the control that a randomized controlled trial brings with it, including the elimination of possibly spurious variables that hide the findings predicted by our hypotheses. Despite these limitations, the present research has made a novel finding, demonstrating for the first time that imaginative engagement, more than framing a piece of music, can have the greatest positive impact on unusual music.

Framing constantly surrounds our everyday music listening experiences, yet opinions remain divided on how it can best be used to facilitate preference. The benefits of progress in this area cannot be understated. Possible implications may include an overall change in the ways that performance venues, music education contexts (such as schools and educational programs at concert halls), radio stations, and the like present and promote music to their audiences and may also influence changes in other creative mediums such as visual art, poetry, dance, film, and television. Based on the studies presented here and our surprising finding that imagination can help to create a positive evaluation of unusual music, the role of framing upon music preference plays a role quite likely larger than current empirical research acknowledges.

## Data Availability Statement

The datasets generated for this study will not be made publicly available as the ethics approval terms state that no personal data is to be made publicly available.

## Ethics Statement

The studies involving human participants were reviewed and approved by the University of New South Wales Human Ethics Committee (HC13015). All participants provided their written informed consent to take part in this study.

## Author Contributions

AC designed, collected, and analyzed the data as well as drafted and refined the manuscript. ES had overall oversight of the project and worked with AC in refining issues concerned with design, analysis, and manuscript writing. Both authors contributed to the article and approved the submitted version.

## Conflict of Interest

The authors declare that the research was conducted in the absence of any commercial or financial relationships that could be construed as a potential conflict of interest.

## Footnotes

1 We refer to the “abstractness” or “atypicality” of the stimulus as “unusualness”, characterized by extremely high complexity, extremely high unfamiliarity, extremely high puzzlingness, and so on, for the listener [see Chmiel and Schubert (2018, 2019b)]. Henceforth, the term “unusual” will be used to describe the term “atypical”, although at times the term “abstract” will be retained for use in visual art due to the prevalence of the term to describe stylistic components in this medium.

2 A study by Margulis et al. (2017) was excluded on the basis that framing was manipulated in a manner that did not vary the perception of quality or an equivalent. Music excerpts were instead accompanied by a short description of the composer’s intention, which focused on the underlying valence of the emotional tone of the work (positive, negative, or ambiguous).

3 Zalanowski (1986) is reviewed in Chmiel and Schubert (2019a), although it did not meet the inclusion criteria for the literature review in study 1 as quality was not manipulated.

4 Kruskal–Wallis *H* tests compare the median ratings between independent variable levels as opposed to the mean ratings. Figures 4, 5 depict the mean ratings with error bars.
