# Supplementary material for: Imaginative Enrichment Produces Higher Preference for Unusual Music Than Historical Framing: A Literature Review and Two Empirical Studies
Source: Front Psychol. 2020 Aug 21;11:1920. doi: 10.3389/fpsyg.2020.01920 (PMC7473496; doi:10.3389/fpsyg.2020.01920)
Supplement: Supplementary file 1 [file Data_Sheet_1.pdf]

Supplementary Material

Imaginative enrichment produces higher preference for unusual music than historical framing: a literature review and two empirical studies

doi: 10.3389/fpsyg.2020.01920

Anthony Chmiel[a], Emery Schubert[b,*]

[a]The MARCS Institute for Brain, Behaviour, and Development, Western Sydney University, Sydney, NSW, Australia
[b]Empirical Musicology Laboratory, School of the Arts and Media, University of New South Wales, Sydney, NSW, Australia

*** Correspondence:**
Emery Schubert
e.schubert@unsw.edu.au

The following material was collated in this supplementary document to avoid an overly lengthy main document.

CONTENTS

1 STUDY 2 SUPPLEMENTARY MATERIAL 2

Supplementary Table 1 3

2 STUDY 3 SUPPLEMENTARY MATERIAL 3

Supplementary Table 2 5

1 Study 2 Supplementary material

Participants in Study 2 received framing for all stimuli. For the "unusual test stimulus" participants received one of the three types of framing listed below for that piece, whereas for the four "other stimuli" all participants received the same, brief historical framing. Participants in the "Historical positive" or "Historical negative" framing conditions were informed of the title of the test stimulus, the composer, and the year of publication/release, whereas those in the "Imaginative engagement" condition were not.

N.B., while the online survey contained a text box for open-ended responses for those in the Imaginative engagement condition, this was not mandatory for submission of the survey and so is not examined in further detail.

Unusual test stimulus: *Six pieces for orchestra*

Historical positive framing: This is a revolutionary work of the mid-twentieth century, demonstrating a radical, new, innovative way of composing, rejecting overly gratuitous musical writing and allowing the music to be "sounds". The focus of the compositional style is to produce colors with the orchestra, rather than the conventional melodies and harmonies that were expected in most Western musics up until the twentieth century, and all popular musics. When people listen to these pieces as "sound colors" they tend to appreciate the music more. This excerpt contains the first two movements of the work.

Historical negative framing: We are playing this piece because it is in an atonal style, which is part of the modernist movement of the twentieth century that rejected romantic, "easy-to-listen-to" melodies. Numerous music psychology studies have confirmed that this music is difficult to process cognitively, explaining why many people don't like it, and why it not only went out of fashion quickly, but was rarely played in the public arena at all. This is due to the considerable presence of dissonance, however we think that individuals may respond to the piece in a variety of ways. This excerpt contains the first two movements of the work.

Imaginative engagement condition: This piece has been used to accompany a science fiction short film about an eerie, desolate planet inhabited by futuristic robots trying to find some kind of emotional connection and meaning in their artificial lives. Although the film is not available here, we ask you to imagine and guess what the storyline might be by writing a few notes in the text box.

Other stimuli (all participants received the same framing)

Black hole sun: The Brad Mehldau Trio are a post-bop jazz ensemble made up of piano, double bass and percussion. While they perform a mix of their own pieces and jazz standards they also incorporate jazz arrangements of popular music. This piece is taken from their 2008 album "Brad Mehldau Trio Live".

Bohemian rhapsody: Bohemian Rhapsody was released in 1975, and written by Queen frontman Freddie Mercury. This piece has no chorus, but rather consists of several distinct sections. Bohemian Rhapsody held the number one position in the UK charts for nine weeks, and in 2012 the song topped

the list on an ITV nationwide poll in the UK to find “The Nation's Favourite Number One” over 60 years of music.

Kora demonstration: The Kora is a 21-string bridge harp found in various areas of West Africa including Gambia, Mali and Senegal. It was traditionally played at local courts by “griots”, who were travelling musicians somewhat similar to the European bard. The Kora is plucked with two fingers from each hand, and can provide a bass-line, fixed melody and simultaneous improvised melody.

The most unwanted song: This is an excerpt from a piece of music which was created by using the results of a survey, in which respondents were asked which elements of music they enjoyed the least. Some elements incorporated into the piece are bagpipes, harp, a small children’s choir and cowbell.

Supplementary Table 1

Significance values of Bonferroni-corrected post hoc tests for preference ratings between pieces in Study 2 (with the conditions for the test pieces collapsed). These tests followed a within-subjects ANOVA examining preference ratings (dependent variable) between the five pieces (independent variable).

Compared pieces	*p*
Six pieces for orchestra – Black hole sun	<.001
Six pieces for orchestra – Bohemian rhapsody	<.001
Six pieces for orchestra – Kora demonstration	<.001
Six pieces for orchestra – The most unwanted song	<.001
Black hole sun – Bohemian rhapsody	.077
Black hole sun – Kora demonstration	>.999
Black hole sun – The most unwanted song	<.001
Bohemian rhapsody – Kora demonstration	.005
Bohemian rhapsody – The most unwanted song	<.001
Kora demonstration – The most unwanted song	<.001

2 Study 3 Supplementary material

Participants in Study 3 received framing for all stimuli. For the “unusual test stimulus” participants received one of the three types of framing listed below for that piece, and similarly for the “typical test stimulus” participants received one of the three types of framing listed below for that piece. For the three “other stimuli” all participants received the same, brief historical framing. Participants in the “Historical positive” or “Historical negative” framing conditions were informed of the title of the test stimulus, the composer, and the year of publication/release, whereas those in the “Imaginative engagement” condition were not.

N.B., while the online survey contained a text box for open-ended responses for those in the Imaginative engagement condition, this was not mandatory for submission of the survey and so is not examined in further detail.

Unusual test stimulus: *Elevation*

Historical positive framing: This piece falls under the umbrella of "free jazz", an experimental style centering on a departure from the associated features of other jazz styles, which were seen as constraints. Rather than using a fixed tempo, structure and set of chord changes free jazz re-directs the focus on collaborative improvisation from all musicians involved, often with little or no set ideas on what a piece should incorporate before it is spontaneously composed. Pharoah Sanders is one of the figures most prominently associated with free jazz, known for using harsh techniques and a chaotic approach to his compositions.

Historical negative framing: This piece falls under the umbrella of "free jazz", an experimental style centering on a departure from the associated features of other jazz styles, which were seen as constraints. Rather than using a fixed tempo, structure and set of chord changes free jazz re-directs the focus on collaborative improvisation from all musicians involved. The chaotic approach and harsh techniques associated with the style have helped to direct the music toward a very niche market, however sales and performance statistics indicate that this particular artist was not received well by most free-jazz listeners.

Imaginative engagement condition: This piece is from a performance style in which large images are projected for the artists and audience to view, and the emotions depicted on these images are intended to lead the direction of the pieces. The soloists improvise on the instruments of other members, regardless of how little experience or knowledge they have on their designated instrument. Although the images are not available here, we ask you to guess what the images and/or emotions might be by writing a few notes in the text box.

Typical test stimulus: *Allegro burlesque*

Historical positive framing: Friedrich Kuhlau was a Danish composer, born in Germany in 1786 and dying in Copenhagen in 1832. He was a known concert pianist and composer throughout Scandinavia. Kuhlau knew Beethoven personally and was influenced by his music, as this short movement demonstrates. It is the "Allegro burlesque" from the third of a set of four Sonatinas, Op. 88, composed and published in 1827, the year of Beethoven's death.

Historical negative framing: Friedrich Kuhlau was a Danish composer, born in Germany in 1786 and dying in Copenhagen in 1832. His work was not taken seriously because of its weak structural aspects, lack of originality and attempt to be overly popular. While some find his tunes catchy, critics of the day reported his music as boring and repetitive. Despite being born in the same country and being a contemporary of Beethoven, the famous master was known to keep a good distance from Kuhlau. This piece is one of Kuhlau's better known works, but is frequently used to accompany comical skits in film and live shows. It is degradingly called the "Allegro burlesque".

Imaginative engagement condition: Imagine a silent film with the following accompanying piece. In the description answer box, we ask you now to make up a story about the piece - the narrative or scene that you might expect the music to accompany. For example, a kidnapped damsel who is saved by a hero, or a slapstick comedy with a sad twist. Be as imaginative and creative as you like.

Other stimuli (all participants received the same framing)

Ain't no mountain high enough: This song was originally recorded in 1967 by Marvin Gaye and Tammi Terrell, and was a Top 20 hit. The version featured today is the original, although numerous re-recordings have emerged since. Amongst others, Diana Ross released a cover of the piece in 1970, which became her first number 1 hit, and Lauryn Hill recorded a more upbeat version for the movie *Sister Act 2* in 1993.

The drunken concubine: Peking Opera, or Beijing Opera, is a form of traditional Chinese theatre that combines music, vocal performance, mime, dance and acrobatics. The style of music arose in the late 18th Century, where it was extremely popular in the Qing Dynasty court.

Thrift shop: "Thrift shop" is a collaboration between rapper Macklemore and producer Ryan Lewis, which has sold over 5 million copies in the USA since its debut in October 2012. In Australia, Thrift shop spent seven weeks in the number one slot, while similarly it held the number one spot in the US Charts for six weeks until being pushed down to number two.

Supplementary Table 2

Significance values of Bonferroni-corrected post hoc tests for preference ratings between pieces in Study 3 (with the conditions for the test pieces collapsed). These tests followed a within-subjects ANOVA examining preference ratings (dependent variable) between the five pieces (independent variable).

Compared pieces	*p*
Elevation – Allegro burlesque	<.001
Elevation – Ain't no mountain	<.001
Elevation – The drunken concubine	>.999
Elevation – Thrift shop	<.001
Allegro burlesque – Ain't no mountain	.007
Allegro burlesque – The drunken concubine	<.001
Allegro burlesque – Thrift shop	>.999
Ain't no mountain – The drunken concubine	<.001
Ain't no mountain – Thrift shop	<.001
The drunken concubine – Thrift shop	<.001
